# Supplementary material for: EBV Impact in Peripheral Macrophages’ Polarization Cytokines in Pediatric Patients
Source: Viruses. 2023 Oct 17;15(10):2105. doi: 10.3390/v15102105 (PMC10612087; doi:10.3390/v15102105)
Supplement: Supplementary file 1 [file viruses-15-02105-s001.zip › Suplementary table S1.pdf]

	VCA IgM	VCA IgG 1/10, 1/40, 1/320	EA IgG	EBNA1 IgG
Primary Infected (PI)	+	-/+	-/+	-
Healthy Carrier (HC)	-	+	-	+
Reactivation (R)	-/+	+	+	+
Not Infected (NI)	-	-	-	-

Supplementary Table S1. Infectious status definition by serology. Primary infected patients (PI) were defined as VCA-IgM+/VCA-IgG-/+/EA-IgG-/EBNA1-IgG-; healthy carriers (HC) as VCA-IgM-/VCA-IgG+/EA-IgG-/EBNA1-IgG+; patients undergoing viral reactivation (R) were defined as VCA-IgM+/-/VCA-IgG+/EA-IgG+/EBNA1-IgG+; and non-infected patients (NI) as VCA-IgM-/VCA-IgG-/EA-IgG-/EBNA1-IgG-
