# Supplementary material for: EBV Impact in Peripheral Macrophages’ Polarization Cytokines in Pediatric Patients
Source: Viruses. 2023 Oct 17;15(10):2105. doi: 10.3390/v15102105 (PMC10612087; doi:10.3390/v15102105)
Supplement: Supplementary file 1 [file viruses-15-02105-s001.zip › Suplementary table S2.pdf]

	Sex	Age
Primary Infected (PI)	F	4
	M	12
	M	3
	F	10
	F	10
	F	6
	M	2
	M	8
	M	4
	M	3
	M	3
	F	5
	F	4
	F	11
	F	5
	M	2
	M	6
	M	12
	F	9
	M	8
Healthy Carrier (HC)	M	7
	M	2
	F	7
	F	2
	M	4
	F	6
	F	12
	F	8
	F	15
	M	8
	F	3
	F	5
	M	11
	F	3
	F	4
	M	6
	F	3
	F	13
	M	3
	M	9
	M	7
	M	4
	M	7

Reactivation (R)	M	5
	M	8
	M	2
	M	2
	M	8
	F	5
	F	3
	F	2
	M	6
	M	11
	M	2
Not Infected (NI)	F	1
	F	1
	F	11
	F	2
	M	2

Supplementary Table S2. Detail of Age and sex in infectious status.
