# Supplementary material for: EBV Impact in Peripheral Macrophages’ Polarization Cytokines in Pediatric Patients
Source: Viruses. 2023 Oct 17;15(10):2105. doi: 10.3390/v15102105 (PMC10612087; doi:10.3390/v15102105)
Supplement: Supplementary file 1 [file viruses-15-02105-s001.zip › Suplementary Table S3.pdf]

	EBERs	LMP1	EBNA2	BMRF1
Latency 0	-	-	-	+/-
Latency I	+	-	-	+/-
Latency II	+	+	-	+/-
Latency III	+	+	+	+/-
Lytic Cycle	+/-	+/-	+/-	+

Supplementary Table S3. Latency Patterns definition by IHQ. Latency 0 was defined as: EBERs-/LMP1-/EBNA2-; Latency I as: EBERs+/LMP1-/EBNA2-; Latency II as: EBERs+/LMP1+/EBNA2-; and Latency III as: EBERs+/LMP1+/EBNA2+
