# Supplementary figures and images for: EBV Impact in Peripheral Macrophages’ Polarization Cytokines in Pediatric Patients
Source: Viruses. 2023 Oct 17;15(10):2105. doi: 10.3390/v15102105 (PMC10612087; doi:10.3390/v15102105)

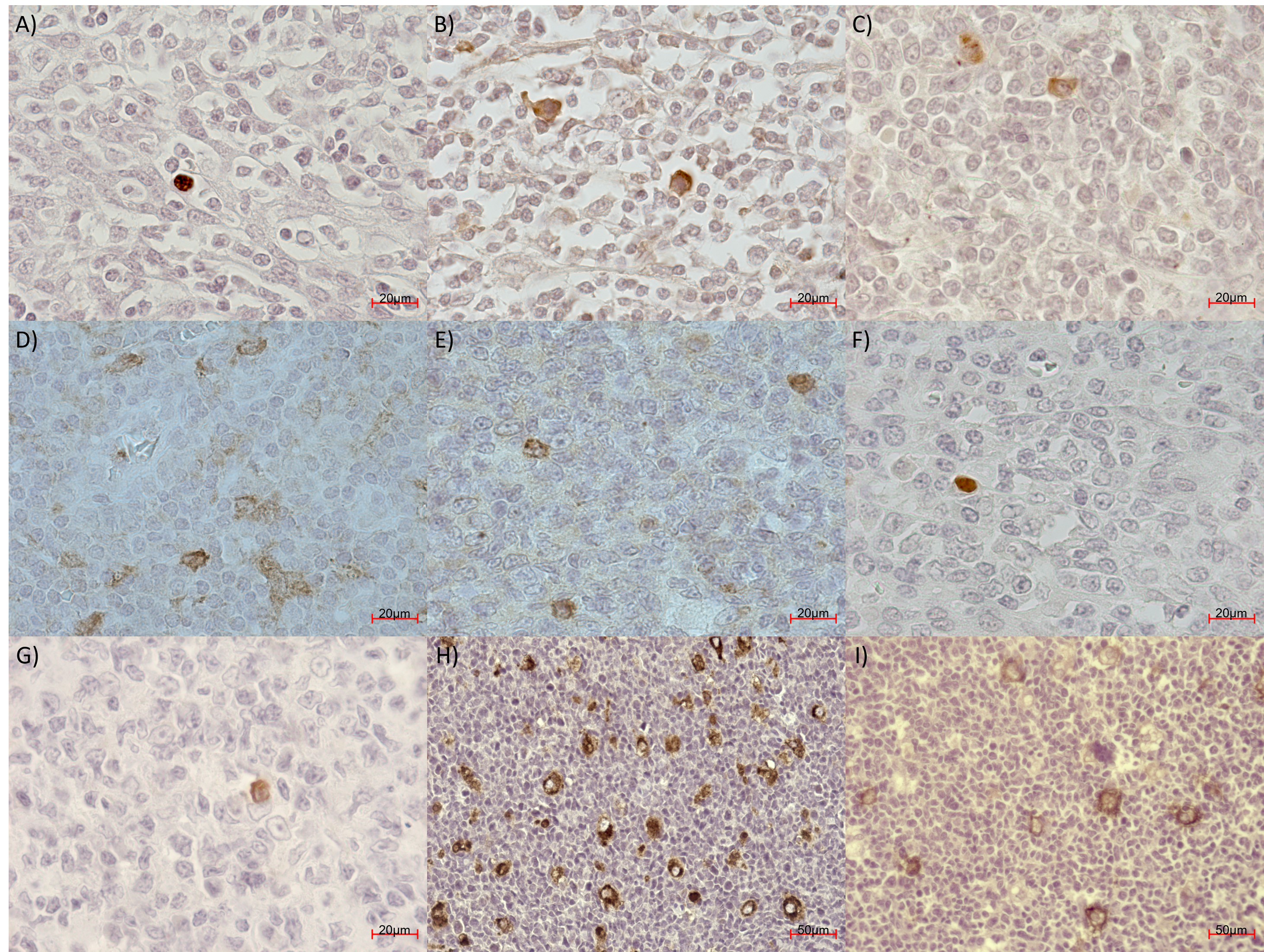

Supplement: Supplementary file 1 [file viruses-15-02105-s001.zip › Supplementary Figure S1.png]
